# Growth of marine fungi on polymeric substrates

**DOI:** 10.1186/s12896-016-0233-5

**Published:** 2016-01-16

**Authors:** Yanming Wang, Dorothee Barth, Anu Tamminen, Marilyn G. Wiebe

**Affiliations:** VTT Technical Research Centre of Finland, P.O. Box 1000, FI-02044 VTT Espoo, Finland

**Keywords:** Marine fungi, Hydrolytic enzymes, Radial growth rate, *Calcarisporium*, *Scopulariopsis*, *Tritirachium*, *Bartalinia*, *Penicillium*, *Pestalotiopsis*

## Abstract

**Background:**

Marine fungi are a diverse group of opportunistic and obligate organisms isolated from marine environments. These fungi are now often included in screens for novel metabolites, while less attention has been given to their production of hydrolytic enzymes. Most enzymes derived from marine microorganisms have been obtained from marine bacteria. The enzymes produced by marine fungi may have different properties than those derived from bacteria or from terrestrial fungi. Here we assess the growth of six filamentous marine fungi on a wide range of polymeric substrates as an indication of their general capacity to produce hydrolytic enzymes.

**Results:**

*Calcarisporium* sp. KF525, *Tritirachium* sp. LF562, *Bartalinia robillardoides* LF550, *Penicillium pinophilum* LF458, *Scopulariopsis brevicaulis* LF580 and *Pestalotiopsis* sp. KF079 all grew on both casein and gelatin as N-source, indicating secretion of proteases. All species also grew on starch, laminarin, xylan, pectin and oil, indicating production of amylases, glucanases, xylanases, pectinases and lipases. Growth on cellulose occurred but was weaker than on xylan. All strains also grew to some extent on sulphated arabinogalactan, although only LF562 could utilise arabinose. Four strains grew on the sulphated ulvans, whereas only KF525 grew on agar or carrageenan. KF525 and LF562 showed limited growth on alginate. Although fucose was used as carbon source by several species, fucoidan did not support biomass production.

**Conclusions:**

Marine fungi could be excellent sources of a wide range of hydrolytic enzymes, including those able to hydrolyse various seaweed polymers. Although the native hosts may secrete only small amounts of these enzymes, the genes may provide a rich source of novel enzymes.

**Electronic supplementary material:**

The online version of this article (doi:10.1186/s12896-016-0233-5) contains supplementary material, which is available to authorized users.

## Background

Marine fungi are a diverse group of opportunistic and obligate organisms isolated from marine environments [1, 2]. In recent years interest in the secondary metabolites and bio-active compounds produced by some of these fungi has grown [2, 3] and they are increasingly included in ecological studies of marine environments [1, 2, 4]. Recent initiatives to develop and exploit marine resources have led to an increase in the isolation of these fungi and screening for novel compounds.

The marine environment provides microorganisms with substrates which may differ considerably from soil, freshwater and plant environments. For example, macroalgae (seaweeds) contain not only compounds like cellulose and xylan, but also a range of polysaccharides not generally observed in land organisms, such as alginate, agar, carrageenan, ulvan, and fucoidan, many of which are sulphated. The polymers may contain monomers of fucose and uronic acids. Although there are accounts of fungi able to degrade these polymers (e.g. [5–7]), most of the research has focused on bacterial enzymes [8]. Since fungi are well known for their ability to produce and secrete proteins, including industrially relevant enzymes, this may reflect a lack of access to marine fungal strains by those carrying out the majority of studies. Marine fungi which have been screened for enzymatic activies have generally been found to produce diverse activities [2].

Of the enzymes from marine fungi which have been studied, cellulases, xylanases and peroxidases have received the most interest [2]. Some of the enzymes identified have unique properties of cold [9], salt [10], alkali [11] or acid [12] tolerance, which may also be of commercial interest.

In this paper we consider the capacity of six diverse marine fungi (*Calcarisporium* sp., *Tritirachium* sp., *Bartalinia robillardoides*, *Penicillium pinophilum*, *Scopulariopsis brevicaulis* and *Pestalotiopsis* sp.) to utilise a range of polymeric substrates derived primarily from plants or macroalgae for growth (Additional file 1: Table S1). Growth on these substrates indicated that they produce a wide range of polymer degrading enzymes, including low levels of enzymes which degrade or modify sulphated polymers. The results confirm that marine fungi could be a good source of enzymes, including enzymes for degradation of macroalgal polymers, which may become more widely available as these are being considered as a source of biomass for biorefineries.

## Results and discussion

**Fig. 1:**
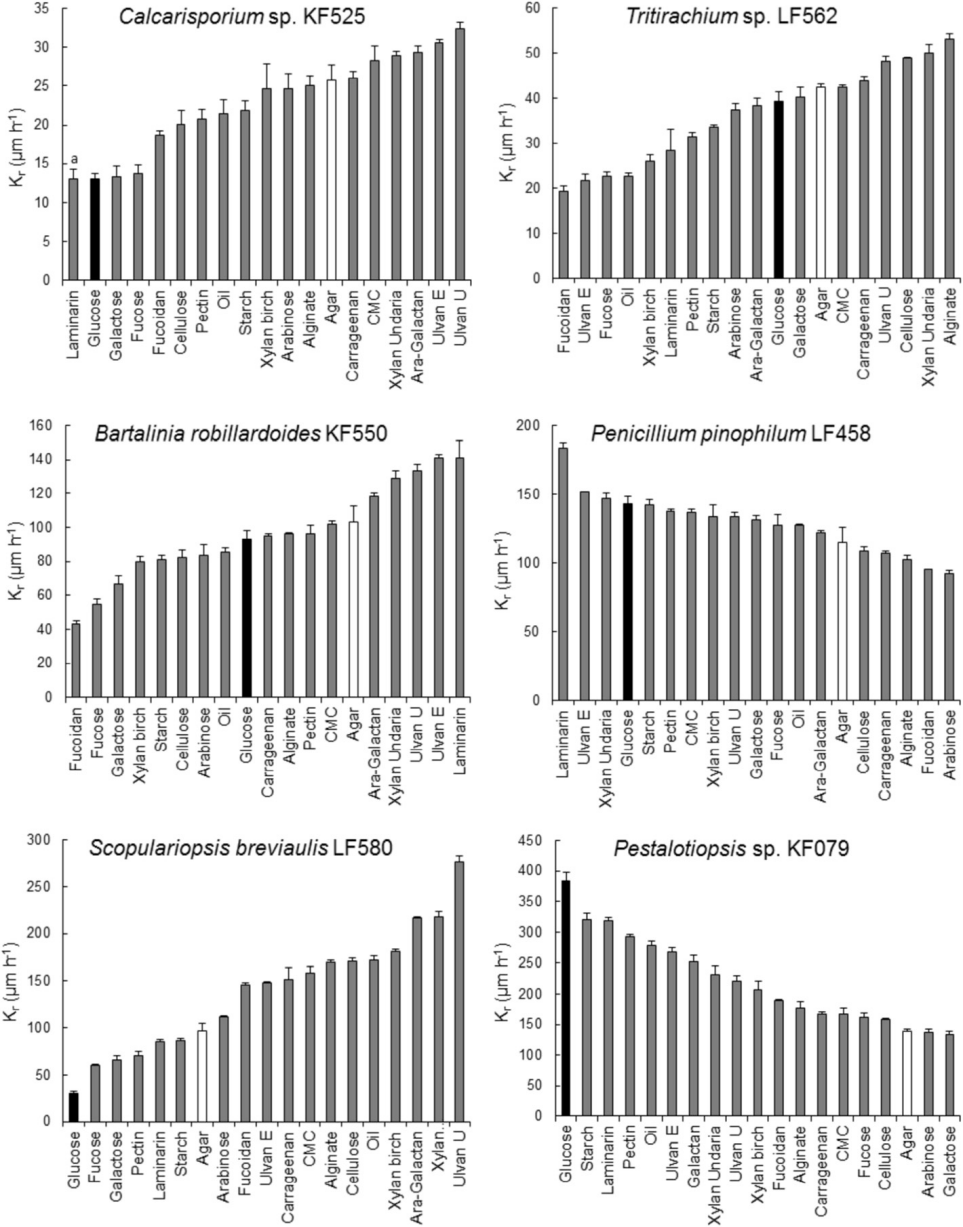
Colony radial growth rates (K_r_) on various carbon sources, ammonium as N source. Fungi were grown on agar-solidified medium containing monomeric (glucose, galactose, arabinose or fucose) or polymeric carbon sources. The colony radial growth rate on glucose is indicated in black and on agar in white. Error bars represent ± sem. Xylan was derived from birch or *U. pinnatifida*. Ara-Galactan = sulphated arabinogalactan from *C. fragile*, Ulvan U = ulvan from *U. armoricana* and Ulvan E = ulvan from *E. intestinalis*

**Fig. 2:**
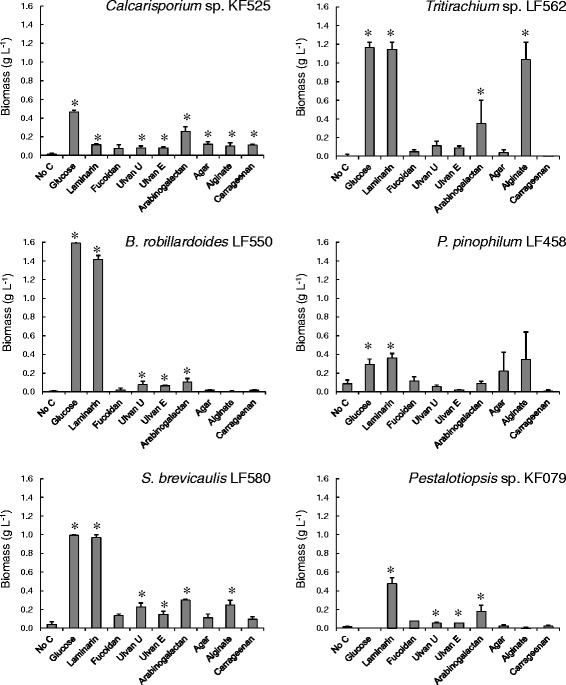
Fungal biomass produced in liquid after 7 days from glucose or macroalgal carbon sources. Fungi were grown in flasks with 2 g L^−1^ of the substrate. Asterisks indicate values which differ significantly (*p* < 0.05) from the amount of biomass present in flasks which received no carbon source. Ulvan U = ulvan from *U. armoricana* and Ulvan E = ulvan from *E. intestinalis*

All strains grew with glucose as sole carbon source and nitrogen provided as ammonium (Table 1). To avoid the decrease in pH associated with uptake of ammonium from the medium, growth on urea was also assessed, but *P. pinophilum* LF458 was unable to use it as a nitrogen source and *B. robillardoides* LF550 grew poorly, so ammonium was used as the nitrogen source for comparing growth on the various carbon substrates. Growth was assessed as colony radial growth rate (K_r_) on the various substrates (Fig. 1, Table 2) and as dry biomass on polymers derived from macroalgae (Fig. 2). It should be noted that colony radial growth rate does not necessarily reflect the amount of biomass produced [13]. Some strains branch less frequently on poor carbon sources, enabling the hyphae to extend more rapidly in search of better carbon sources. Thus growth (sparse or dense colonies) on agar-solidified medium was also assessed visually (Table 1). All strains grew to some extent on media containing only agar as carbon source or lacking nitrogen (Tables 1 and 2), which was taken into consideration when assessing growth on other substrates, such as arabinose.

**Table 1:** Growth on agar-solidified medium with various carbon sources, ammonium as N source

Strain^a^	KF525	LF562	LF550	LF458	LF580	KF079
Glucose	++++^b^	++++	++++	++++	++++	++++
Laminarin	++++	++++	++++	++++	++++	++++
Starch	++++	++++	++++	++++	++++	++++
Cellulose	+++	+++	+++	++	+++	+++
CMC	++	++	+++	++	+++	+++
Xylan (birch)	+++	++++	++	+++	+++	+++
Xylan (*Undaria*)	++++	++++	++++	++++	++++	++++
Pectin	++++	++++	++++	++++	++++	++++
Oil	+++	+++	++++	++	+++	++++
Ulvan (*Ulva*)	++++	+++	++	++	+	++
Ulvan (*Enteromorpha*)	++++	+++	++	++	+	+++
Arabinogalactan	++++	+++	++	++	++	+++
Galactose	++++	++++	+++	++++	++++	++++
Arabinose	++	++++	+	+	+	++
Fucoidan	+++	+++	++	++	+	++
Fucose	+++	++++	+++	++	++	++
Alginate	+++	+++	++	++	++	++
Carrageenan	++	++	++	++	++	++
Agar (no C)	++	++	+	+	+	++

^a^ *Calcarisporium* sp. KF525, *Tritirachium* sp. LF562, *Bartalinia robillardoides* LF550, *P. pinophilum* LF458, *S. brevicaulis* LF580 and *Pestalotiopsis* sp. KF079

^b^Extent of growth indicated as: + weak, ++ moderate, +++ good or ++++ very good

**Table 2:** K_r_ and growth on agar-solidified medium with various N sources, glucose as C source

Strain^g^	KF525	LF562	LF550	LF458	LF580	KF079
Ammonium	13 ± 1^ea^	40 ± 2^b^	93 ± 5^b^	143 ± 5^d^	30 ± 3^a^	384 ± 15^d^
	++++^f^	++++	++++	++++	++++	++++
Nitrate	16 ± 1^b^	41 ± 1^b^	60 ± 3^a^	98 ± 2^a^	134 ± 2^c^	228 ± 4^b^
	+	++++	+++	++++	++++	++++
Urea	16 ± 2^b^	40 ± 1^b^	54 ± 2^a^	110 ± 1^ab^	103 ± 2^b^	264 ± 3^c^
	+++	++++	++	+	+++	+++
Casein	21 ± 2^b^	49 ± 0^c^	94 ± 2^b^	127 ± 2^c^	198 ± 9^d^	106 ± 2^a^
	+++	++++	+++	+++	++++	+++
Gelatin	27 ± 1^c^	49 ± 2^c^	84 ± 4^b^	115 ± 3^b^	212 ± 3^d^	236 ± 8^bc^
	+++	++++	+++	++	+++	+++
No nitrogen	15 ± 1^ab^	34 ± 1^a^	63 ± 3^a^	119 ± 1^a^	99 ± 5^b^	256 ± 7^c^
	+	++	+	+	++	++

^e^Values are average K_r_ ± sem (μm h^−1^) for n = 4 to 12. Values for the same strain with the same superscript letter (a to d) did not differ significantly (*p* > 0.05)

^f^Extent of growth indicated as: + weak, ++ moderate, +++ good or ++++ very good

^g^ *Calcarisporium* sp. KF525, *Tritirachium* sp. LF562, *Bartalinia robillardoides* LF550, *P. pinophilum* LF458, *S. brevicaulis* LF580 and *Pestalotiopsis* sp. KF079

All strains grew on both casein and gelatin as nitrogen sources, indicating that all strains produced extracellular proteases (Table 2). *Calcarisporium* sp. KF525, *Tritirachium* sp. LF562, *S. brevicaulis* LF580, and *Pestalotiopsis* sp. KF079 caused precipitation of para-κ-casein from casein, whereas *B. robillardoides* LF550 and *P. pinophilum* LF458 did not. Growth on casein was generally better than growth on gelatin, but LF580 and LF562 grew equally on both protein sources. Good protease production has also been observed from marine derived *Aspergillus*, *Beauveria* and *Acremonium* species [14]. All strains except *Calcarisporium* sp. KF525 were able to use nitrate as sole nitrogen source (Table 2).

### Growth of *Calcarisporium* sp. KF525 on polymeric carbon sources

*Calcarisporium* species are commonly isolated as mycoparasites or symbionts of higher fungi [15], and less frequently from marine environments. Strain KF525 was isolated from the German Wadden Sea and has been shown to produce macrocyclic and linear polyesters and cyclodepsipeptides [16, 17]. Parasitic species are also known to produce various bioactive compounds [18–21]. The variability of intracellular enzymes of some strains of *C. arbuscula* has been assessed [22]. The physiology of strain KF525 has not been extensively characterised, but Tamminen et al. [23] reported that it grew slowly (μ = 0.03 - 0.06 h^–1^) and did not grow at temperatures above 24 °C.

*Calcarisporium* sp. KF525 grew on agar-solidified medium on all carbon sources provided except perhaps arabinose, carboxymethyl cellulose (CMC) and carrageenan, on which growth was similar to that observed on agar (Table 1, Fig. 1). It produced sparser, more rapidly extending hyphae in the absence of easily metabolised carbon source than in its presence (Fig. 1). Thus low K_r_ values for KF525 represent better growth than higher K_r_ values and glucose, laminarin, galactose and fucose represented the most readily utilised carbon sources (Kr ~ 13 μm h^−1^; Fig. 1, Table 1). Cellulose, pectin, oil, starch, and xylan (from either birch or from the macroalga *Undaria pinnatifida*) also supported good biomass production, although the K_r_ values were higher than on glucose. Growth on ulvans, fucoidan and alginate was more dense than that observed when agar was the only C-source or with agar and arabinose or carrageenan, although the K_r_ on ulvans was high (Fig. 1).

KF525 produced high background growth on medium containing only agar, suggesting that it was able to partially utilise agar as a carbon source. This was confirmed by growing KF525 in flasks in medium containing 2 g L^−1^ agar as the sole carbon source. Growth was observed within 7 days (0.12 ± 0.02 g l^−1^), whereas essentially no growth (0.01 ± 0.01 g L^−1^) had occurred in medium lacking a carbon source (Fig. 2). Similarly, KF525 grew on carrageenan (0.11 ± 0.01 g L^−1^), but did not completely metabolise it. Growth on ulvan (from either *Ulva* or *Enteromorpha*, 0.08 ± 0.01 g L^−1^), arabinogalactan (0.26 ± 0.05 g L^−1^), and alginate (OD or DW) in the absence of agar was also confirmed in liquid cultures. KF525 produced 0.47 ± 0.02 g l^−1^ biomass from glucose and 0.12 ± 0.01 g L^−1^ from laminarin during the same time interval.

KF525 showed the most extensive growth on substrates such as agar, carrageenan and alginates, of the fungi considered here. It also grew on sulphated ulvans and arabinogalactans, although it did not appear to utilise arabinose. The slow growth of KF525 would be undesirable in a production organism, but it may be a source of novel fungal enzymes which could be expressed in alternative hosts.

### Growth of *Tritirachium* sp. LF562 on polymeric carbon sources

*Tritirachium* sp. LF562 was isolated from a sponge growing in the Adriatic Sea [24]. *Tritirachium* species have been described as airborne contaminants and have been isolated from plant and packing material [25] and from soil [26, 27]. *T. album limber* is a producer of serine protease [27, 28]. Although not common in the marine environment, *T. candoliense* has also been isolated from the anoxic zone in the Arabian Sea [29] and others have been isolated from Australian coastal waters [30]. Apart from the proteinases, hydrolytic enzymes have not been studied.

LF562 had a low K_r_ (19 to 53 μm h^−1^), changes in which did not reflect either good or poor growth (Fig. 1). Better growth was observed on galactose, fucose, arabinose, starch, laminarin, xylan (clearing zones present after 1 day of incubation), pectin and oil (Table 1). LF562 was the only strain included in this study which utilised arabinose as a carbon source. LF562 also grew on cellulose, and to some extent on CMC, ulvans, arabinogalactan, fucoidan, and alginate.

LF562 grew as well on laminarin as on glucose in liquid culture (Fig. 2), with a yield of 0.57 g biomass per g substrate consumed. Biomass production on arabinogalactan (0.35 ± 0.25 g L^−1^) and alginate (1.04 ± 0.19 g L^−1^) was also confirmed in liquid cultures. Biomass production from ulvans (0.12 ± 0.05 and 0.09 ± 0.02 g L^−1^) was comparable to that of KF525 or LF550, but was not significantly greater (*p* > 0.05) than that of the no carbon control because of variability between the cultures.

### Growth of *Bartalinia robillardoides* LF550 on polymeric carbon sources

*Bartalinia robillardoides* LF550 was isolated from a marine sponge (*Tethya aurantium*) obtained from the Adriatic Sea near Rovinj, Croatia [24]. A *Bartalinia* specie has also been isolated from the sponge *Gelliode fibrosa* in the Pacific [31], but *B. robillardoides* strains are more typically isolated from plants [32, 33], leaf litter [34] or insect [32]. Apart from taxonomic studies, the main interest in *B. robillardoides* has been in its secondary metabolites, which include taxol [33] and various azophilones [35]. Enzyme activities from *B. robillardoides* have not been reported.

LF550 grew well on glucose, laminarin, starch, xylan (particularly that derived from the macroalga *Undaria*, clearing zones present after 1 day of incubation), pectin, oil, cellulose, CMC, galactose, and fucose (Table 1). Weak growth was observed on ulvans, arabinogalactan and fucoidan (Table 1). There was no clear correlation between K_r_ and good growth. Low K_r_s on fucoidan and fucose indicated that these substrates were perceived by the fungus. LF550 did not grow on arabinose and background growth on agar was very low. Growth on ulvans (0.08 ± 0.04 and 0.07 ± 0.01 g L^−1^) and arabinogalactan (0.11 ± 0.04 g L^−1^) was confirmed in liquid cultures, as was the inability to grow on agar (Fig. 2).

### Growth of *Penicillium pinophilum* LF458 on polymeric carbon sources

*Penicillium* species, including *P. pinophilum*, are frequently isolated from marine environments [24, 30, 36], as well as being common in soil and other land environments. *P. pinophilum*, like other penicillia, is known for producing bioactive metabolites [36, 37]. Enzymes involved in the degradation of lignocellulosic biomass have been identified from various isolates [38–42], and a gene encoding a dextranase was recently cloned from a marine isolate [43].

Like KF079, LF458 generally showed reduced rates of expansion on poor carbon sources, although Kr was not strongly affected by the carbon source (Fig. 1). Dense growth was observed on galactose, starch, laminarin, xylan (clearing zones present after 1 day of incubation), and pectin (Table 1). Weak growth was observed on cellulose, CMC, oil, ulvans, arabinogalactan, fucose, fucoidan, alginate and carrageenan. Background growth on agar was very low, as was growth on arabinose. Utilisation of ulvans, fucoidan, alginate, agar or carrageenan was not observed in liquid culture, in which LF458 grew poorly as pellets, with high variation between cultures (Fig. 2). Even in glucose (0.29 ± 0.06 g L^−1^) and laminarin (0.36 ± 0.05 g L^−1^) containing medium, biomass production was low even though all glucose had been consumed.

### Growth of *Scopulariopsis brevicaulis* LF580 on polymeric carbon sources

*Scopulariopsis brevicaulis* is the anamorph of *Microascus brevicaulis* and is commonly found in soil environments. Some strains have been isolated from skin or nails, where it appears to be an opportunistic pathogen. It has only occasionally been isolated from marine environments [44–46], but has also been found in other high salt environments [47, 48]. Production of keratinase [49] and L-methioninase [50] has been reported.

Like KF525, LF580 produced sparser, more rapidly extending hyphae in the absence of easily metabolised carbon source than on glucose (Fig. 1). However, colonies produced on glucose were much more compact (low K_r_) than those growing on fucose, galactose, pectin, laminarin, starch or xylan (clearing zones present after 1 day of incubation), although these substrates also supported good biomass production (Table 1). Thus high K_r_ values for LF580 do not necessarily represent poor growth. Colony expansion on glucose may have been affected by reduction in the local pH (pH ~4 at the edge of colonies grown on glucose with ammonium, while remaining close to 5.5 at the edge of the plate), caused by ammonium uptake in the poorly buffered medium. This hypothesis was supported by the much higher colony expansion rates observed on medium containing either urea (pH ~5 at edge of colony) or nitrate (pH ~8 at colony edge) (Table 2). However, this reduction in pH did not appear to affect growth in liquid medium, in which glucose was completely metabolised (Fig. 2). LF580 also grew on oil, cellulose, CMC and arabinogalactan, whereas growth on carrageenan, alginate or fucose was poor, with even less growth on arabinose, ulvans, fucoidan or agar (Table 1). LF580 colonies expanded much more rapidly on these carbon sources than on agar, indicating that they were sensed by the fungus.

The genome of *S. brevicaulis* LF580 was recently published and the profile of putative carbohydrate-active enzymes identified in the genome analysis [51]. Kumar et al. [51] concluded that LF580 should be well adapted for the breakdown of terrestrial plant materials, having numerous putative glycoside hydrolases with homology to xylanases, pectinases, cellulases and lytic polysaccharide mono-oxygenases. The good growth on xylan and pectin supported their conclusions, although growth on cellulose was weaker than might have been expected.

LF580 produced less background growth on agar-solidified medium lacking other carbon source than KF525 or LF562. Growth in flasks containing 2 g L^−1^ agar as the sole carbon source was not significantly greater (*p* > 0.05) than in flasks without carbon source (Fig. 2). However, measureable growth (*p* < 0.05) was observed on alginate (0.25 ± 0.05 g L^−1^) and arabinogalactan (0.3 ± 0.01 g L^−1^). More biomass was produced from ulvans (0.15 ± 0.04 and 0.23 ± 0.04 g L^−1^) than was expected based on the growth on agar-solidified medium. Biomass production from laminarin (0.97 ± 0.03 g L^−1^) was similar to that from glucose (1.00 ± 0.01 g L^−1^), with a yield of 0.49 g biomass per g substrate indicating complete metabolism (Fig. 2). However, as predicted [51], LF580 appears better adapted for hydrolysis of plant than of macroalgal biomass.

### Growth of *Pestalotiopsis* sp. KF079 on polymeric carbon sources

*Pestalotiopsis* species are predominantly anamorphic, commonly found as endophytes or plant pathogens, but also able to live saprophytically, and are isolated from very diverse environments, including marine environments [52, 53]. They have recently gained considerable interest because of the diversity of secondary metabolites of potential commercial interest which they produce [53]. The cellulases, hemicellulases and laccases of some strains isolated from mangrove swamps have also been investigated [54, 55].

KF079 had a high K_r_ on glucose (384 ± 19 μm h^−1^) and generally showed reduced rates of expansion on poor carbon sources (Fig. 1). Dense growth at high K_r_ values was observed on starch, laminarin, pectin and oil, while dense growth with lower colony expansion rates was observed on xylan (clearing zones present after 1 day of incubation), cellulose, CMC and galactose (Fig. 1). Weak growth was observed on ulvans, arabinogalactan, fucoidan, fucose, arabinose, alginate and carrageenan (Table 1). Background growth on agar was relatively high, but measurable biomass production was not observed within 7 days in shaken flasks containing 2 g l^−1^ agar (0.03 ± 0.01 g l^−1^, compared to 0.02 ± 0.01 g l^−1^ without added carbon, Fig. 2). No biomass was produced from alginate or carrageenan in liquid culture, but growth of KF079 on ulvans (either from *Ulva* or *Enteromorpha*, 0.05 ± 0.00 g l^−1^), fucoidan (0.08 g l^−1^) and arabinogalactan (0.18 ± 0.07 g l^−1^) was observed. Laminarin (0.48 ± 0.06 g l^−1^) was used as the control. KF079 thus appeared more likely to be a source of enzymes for hydrolysing plant biomass than for seaweed-derived biomass, although its ability to utilise ulvans may be of interest.

## Conclusions

Growth of *Calcarisporium* sp. KF525, *Tritirachium* sp. LF562, *Bartalinia robillardoides* LF550, *Penicillium pinophilum* LF458, *Scopulariopsis brevicaulis* LF580 and *Pestalotiopsis* sp. KF079 on polymeric substrates demonstrated that each was able to produce protease, amylase, glucanase, xylanase, pectinases and lipase. Growth on laminarin and starch was generally comparable to that on glucose, demonstrating the efficiency with which amylases and glucanases were produced. Although they grew well on xylan, none of these cells grew well on cellulose or CMC, indicating only weak production of cellulase or endoglucanase.

All strains except *P. pinophilum* LF458 were also able to grow on sulphated arabinogalactan, even though only *Tritirachium* sp. LF562 could metabolise arabinose. All strains utilised galactose. Only *Calcarisporium* sp. KF525 produced biomass from the sulphated galactans, agar and carrageenan. Agarases and carageenases from bacteria have received considerable attention [8], but there are few reports of these activities from filamentous fungi [5].

The sulphated fucoidan was not clearly metabolised by any strain, although they appeared able to use fucose as a carbon source and all responded to the presence of fucoidan in the medium.

Several strains grew on both sources of ulvan, indicating that they may be a source for novel ulvan and glucuronan lyases or hydrolases, few of which have been characterised [56–59]. However, the limited growth observed on ulvan suggests that the enzyme levels are low, or that these strains do not have complete pathways for its degradation.

*Calcarisporium* sp. KF525, *S. brevicaulis* LF580 and *Tritirachium* sp. LF562 produced biomass from alginate. Although most studies of alginate lyases have focused on those obtained from marine bacteria [8], alginate lyase activity has also been observed in marine isolates of *A. oryzae* [6] and *Dendryphiella salina* [7].

Marine fungi clearly produce a wide range of hydrolytic activities and should be considered as more than just providers of novel lignocellulolytic enzymes. More in depth study of the enzymes from marine fungi, as well as from bacteria, is needed to identify novel activities and enzymes with novel properties to enhance our understanding of polymer metabolism and to provide catalysts for future biorefineries. Although the native hosts may secrete only small amounts of these enzymes, their genes may provide a rich new source of novel enzymes.

## Methods

### Strains

*Calcarisporium* sp. KF525, *Tritirachium* sp. LF562, *Bartalinia robillardoides* LF550, *Penicillium pinophilum* LF458, *Scopulariopsis brevicaulis* LF580 and *Pestalotiopsis* sp. KF079 were obtained from the culture collection of the Kiel Center for marine natural products at GEOMAR, Helmholtz Centre for Ocean Research Kiel, as a kind gift from A. Labes and J. F. Imhoff. Stock cultures were maintained as conidia or mycelial fragments suspended in 20 % v/v glycerol, 0.8 % w/v NaCl with ~0.025 % v/v Tween 20 or on Microbank™ Bacterial and Fungal Preservation System beads (Pro-Lab Diagnostics, UK) at −80 °C. Spores of *Calcarisporium* sp. KF525 were obtained from cultures growing in shaken flasks on Yeast Malt Peptone (YMP, 3 g L^−1^ yeast extract, 3 g L^−1^ malt extract, 5 g L^−1^ peptone) medium containing 30 g L^−1^ Sea Salt (Tropic Marin®, Germany) for 11 days, after removal of the mycelia by filtration through cotton. Spores of *Penicillium pinophilum* LF458 and *Scopulariopsis brevicaulis* LF580 were obtained from cultures growing on agar-solidified (15 g L^−1^ agar) YMP medium containing 30 g Tropic Marin® Sea Salt L^−1^. *Tritirachium* sp. LF562, *Bartalinia robillardoides* LF550 and *Pestalotiopsis* sp. KF079 sporulated poorly.

### Media

The defined medium (initial pH ~5.5) contained 0.5 g L^−1^ KH_2_PO_4_, 0.1 g L^−1^ MgSO_4_ · 7H_2_O, 0.05 g L^−1^ CaCl_2_ · 2H_2_O, 5 mg L^−1^ Citric acid · H_2_O, 5 mg L^−1^ ZnSO_4_ · 7H_2_O, 1 mg L^−1^ Fe(NH_4_)_2_(SO_4_)_2_ · 6H_2_O, 50 μg L^−1^ MnSO_4_ · 4H_2_O, 260 μg L^−1^ CuSO_4_ · 5H_2_O, 50 μg L^−1^ H_3_BO_3_, 50 μg L^−1^ Na_2_MoO_4_ · 2H_2_O, and 50 μg L^−1^ biotin. Ammonium sulphate (3.3 g L^−1^), sodium nitrate (4.25 g L^−1^), urea (1.5 g L^−1^), casein (4 g L^−1^) or gelatin (4 g L^−1^) were provided as nitrogen source. Glucose (2 or 10 g L^−1^), fucose, galactose, arabinose, starch, pectin from citrus, cellulose, carboxy-methyl cellulose (CMC), laminarin (from *Laminaria digitata*, Sigma-Aldrich), xylan (from birch, Sigma-Aldrich, or from *Undaria pinnatifida*, Elicityl SA), ulvan (from *Ulva armoricana* or *Enteromorpha intestinalis*, Elicityl SA), sulphated arabinogalactan (from *Codium fragile*, Elicityl SA), carrageenan (from *Eucheuma cottonii*, Sigma-Aldrich), agar (noble, Difco), sodium alginate (from *Macrocystis pyrifera*, Sigma-Aldrich), or fucoidan (from *Fucus vesiculosus,* Sigma-Aldrich) (10 g L^-1^ except for polymers derived from macroalgae, which were 2 g L^−1^) were provided as primary carbon sources. Carbon sources, casein and gelatin were sterilised by autoclaving in water or water containing agar (pH 7) at 121 °C for 15 to 20 min. Salts and inorganic N-sources were sterilised by filtration and added as a concentrated solution to the sterile carbon sources. Agar solidified media contained 15 g L^−1^ agar. All media contained 30 g Tropic Marin® Sea Salt L^−1^ (prepared as a 3-fold concentrated stock, sterilised by filtration). The composition (monomers and their linkage) of polymeric substrates is described in Additional file 1: Table S1.

### Cultural conditions and measurements of growth

Flasks (50 or 100 mL, containing 10 mL medium) were inoculated with conidial suspensions or with approximately 2 x 2 mm^2^ pieces of mycelium, excised from YMP medium and from which excess agar had been removed, and incubated at 22 °C (*Calcarisporium* sp. KF525) or 30 °C (all other strains), 180 rpm.

Agar-solidified medium (in 4.5 or 9 cm diameter Petri dishes) was inoculated centrally with 2 μl spore suspension or with approximately 2 x 2 mm^2^ pieces of mycelium, excised from YMP medium and from which excess agar had been removed. Petri dishes were incubated inverted at 22 °C (KF525) or 30 °C (all other strains).

Colony radial expansion rates (K_r_) were calculated from measurements of colony diameter at 24 to 48 h intervals. Biomass from liquid cultures was collected by filtration (Millipore 0.22 μm GSWP filters or Whatman filter paper 1), washed twice with excess water and taken to dryness at 65 °C. To remove agar, alginate and carrageenan, the culture was warmed at 100 °C then washed twice in excess hot (100 °C) water, which was removed by centrifugation, before collecting the biomass by filtration. This heat treatment may cause some cell lysis and result in an underestimate of the fungal biomass, but was preferable to overestimating the biomass by failing to remove the polymers, which could lead to false conclusions about consumption of the polymer.

The presence or absence of glucose in flask cultures was assessed using Roche Keto-Diabur-Test 5000 sticks, which show green colour changes in the presence of low concentrations (<1 g L^−1^) of glucose. The pH of agar at the edge of colonies was estimated using Merck Acilit® (pH 0–6) or Alkalit® (pH 7.5–14) pH indicator sticks.

## Additional file

Additional file 1: Table S1. Characteristics of polymeric substrates used in this study. (DOCX 22 kb)
