# Supplementary material for: Growth of marine fungi on polymeric substrates
Source: BMC Biotechnol. 2016 Jan 16;16:3. doi: 10.1186/s12896-016-0233-5 (PMC4715362; doi:10.1186/s12896-016-0233-5)
Supplement: Additional file 1: Table S1. — Characteristics of polymeric substrates used in this study. (DOCX 22 kb) [file 12896_2016_233_MOESM1_ESM.docx]

**Additional file 1: Table S1.** Characteristics of polymeric substrates used in this study.

| **Polymer** | **primary monomer(s)** | **other monomers** | **linear linkage** | **branch linkage** | **SO_4_^-^** |
| --- | --- | --- | --- | --- | --- |
| Laminarin | d-glucose | manitol | β(1,3) | β(1,6) | no |
| Starch | d-glucose |  | α(1,4) | α(1,6) | no |
| Cellulose | d-glucose |  | β(1,4) |  | no |
| CMC | d-glucose | carboxymethyl d-glucose | β(1,4) |  | no |
| Xylan (birch) | d -xylose (>90%) | l-arabinose,  4-O-methylglucuronic acid, acetyl | β(1,4) | α(1,3) or α(1,2) | no |
| Xylan (*Undaria*) | d -xylose (>98%) |  | β(1-3) / β(1-4) ratio 1:4 |  | no |
| Pectin | d -galacturonic acid | d-galactose, l-arabinose d-xylose, l-rhamnose | α(1,4), may be methoxylated or acelated | α(1,2) and others | no |
| Oil | fatty acids, glycerol |  | ester |  | no |
| Ulvan | l-rhamnose, d-glucuronic acid | d-xylose, d-glucose, l-iduronic acid | β(1,4), α(1,4), also  1,3-, 1,3,4-, 1,2,3,4- and 1,2,4- | β(1,2) | yes |
| Arabinogalactan | l-arabinose, d-galactose | d-mannose, d-xylose, d-glucose | β(1,3), may be pyruvylated | β(1,6) | yes |
| Fucoidan | l-fucose | d-galactose, d-mannose, d-xylose and uronic acids | α(1,3), α(1,4) | various | yes |
| Alginate | l-guluronic & d-mannuronic acids |  | β(1-4) |  | no |
| Carrageenan | d-galactose, 3,6-anhydrogalactose |  | α(1,3), β-(1,4) |  | yes |
| Agar | d- or l-galactose, 3,6-anhydro- l-galactose |  | α(1,3), β-(1,4) |  | yes |
| Casein | amino acids |  | amine |  | no |
| Gelatin | amino acids |  | amine |  | no |
